# Epidemiology of AKI: Utilizing Large Databases to Determine the Burden of AKI

**DOI:** 10.1053/j.ackd.2017.05.001

**Published:** 2017-07

**Authors:** Simon Sawhney, Simon D. Fraser

**Affiliations:** Kidney Disease@Farr Collaboration, University of Aberdeen, Aberdeen, UK; and Kidney Disease@Farr Collaboration, University of Southampton, Southampton, UK

**Keywords:** Acute kidney injury, Incidence, Prognosis, Big-data, Quality improvement

## Abstract

Large observational databases linking kidney function and other routine patient health data are increasingly being used to study acute kidney injury (AKI). Routine health care data show an apparent rise in the incidence of population AKI and an increase in acute dialysis. Studies also report an excess in mortality and adverse renal outcomes after AKI, although with variation depending on AKI severity, baseline, definition of renal recovery, and the time point during follow-up. However, differences in data capture, AKI awareness, monitoring, recognition, and clinical practice make comparisons between health care settings and periods difficult. In this review, we describe the growing role of large databases in determining the incidence and prognosis of AKI and evaluating initiatives to improve the quality of care in AKI. Using examples, we illustrate this use of routinely collected health data and discuss the strengths, limitations, and implications for researchers and clinicians.

**Clinical Summary**

• Large database studies show that AKI pervades health care systems, with poor outcomes and an increasing use of renal replacement therapy.
• Risk stratification based on acute kidney injury (AKI) severity, baseline, and recovery to baseline and follow-up time may be helpful to improve post-AKI care, but such an approach would need to be evaluated.
• Temporal and regional comparisons of AKI incidence and prognosis may be biased by changes in AKI awareness, monitoring, recognition, recording, and clinical practice.
• Researchers evaluating quality initiatives using observational data should be careful to capture all who may be affected by policy changes, to evaluate both intended and unintended consequences, and to recognize the limitations of potential confounding.

## Introduction—Why Use Large Databases to Study Acute Kidney Injury?

…if you are going to teach people to make observations, you should show that something wonderful can come from them…

–Richard Feynman, nobel laureate^1^

For over a century, epidemiologists have exploited routinely collected health data to observe the causes and natural history of human diseases; 150 years ago, William Farr pioneered the collection of vital statistics and leveraged them for insight into the cholera epidemic in England. The same approach now forms the basis of the *International Classification of Diseases* (*ICD*) used by health systems worldwide.^2^ In Sweden, for example, data contained within each of 90 national registries can be linked by unique personal identity numbers for use in medical research. Swedish data have been instrumental for developing innovative medical devices, monitoring policy, and improving care quality.^3^ Well-conducted large observational studies using routinely collected health data are crucial in health research in the current era of big data and technologic innovation.^4^ Large observational studies are more capable than trials of determining the true burden of a disease in the “real world.” This makes them valuable resources for evaluating disease incidence, prevalence, prognosis, and responses to policy changes. With the increasing financial pressure to design trials efficiently, routine electronic records also show promise for rapid case finding and inexpensive follow-up.^5^ However, observational evidence from routinely collected data also comes with caveats. There may be unaccounted confounding and results can mislead if not interpreted carefully.^6^ In this review, we describe recent large database studies that have furthered understanding of acute kidney injury (AKI) and discuss the advantages and drawbacks of using routine health data in observational research. We focus on 3 themes that are topical in AKI: the apparent increasing incidence of AKI, the long-term prognosis of AKI, and the role of large databases in AKI care quality.

## AKI—The Growing Awareness of a Global Burden

AKI is common (8-16% of hospital admissions^7^), serious (fourfold increased hospital mortality^8^), and many aspects of its natural history remain uncertain.^9^, ^10^ In the United Kingdom, >1% of health service expenditure is attributed to AKI.^11^ Over 20 years, studies of AKI have moved from detailed descriptions of small but well-characterized cohorts^12^ to large-scale analyses involving thousands of people classified using *ICD* hospital episode codes.^13^, ^14^ Also in the last decade, the former term “acute renal failure” has been replaced with standardized AKI criteria incorporating small changes in creatinine and urine output, such as the “Acute Kidney Injury Network” (AKIN)^15^ and “Kidney Disease: Improving Global Outcomes” (KDIGO) criteria.^16^ In many health systems, kidney function data are increasingly available either in integrated electronic health records or through data linkage. In high-income countries, these developments have led to recognition that even small changes in creatinine carry an adverse prognosis^8^ and that some poor outcomes after AKI are preventable.^17^ The International Society of Nephrology 0by25 initiative has an ambitious aim to prevent all avoidable death from AKI worldwide by 2025.^18^ It carried out a global study over 10 weeks in 2014 to capture a snapshot of the burden of KDIGO-based AKI across 289 centers and 72 countries.^19^ Seven-day mortality in this select snapshot was 10-12% in both high- and low-income countries. Key etiologic risk factors varied between countries but included dehydration, shock, infection, sepsis, cardiac disease, and nephrotoxic medications. The study showcased the potential for global data collection to inform international strategy.

## The Population Incidence of AKI

Numerous studies have exploited *ICD* coding of hospital episodes to quantify the incidence of AKI across a range of clinical settings (Table 1). They consistently report a rising incidence of AKI over time, whether requiring or not requiring renal replacement therapy (RRT).^45^ If real, this represents a major global public health concern, but it is hard to interpret these findings amid changes in AKI definitions, awareness of AKI, and clinical practice.^45^

**Table 1:** Summary of Studies that Have Described Temporal Trends in the Incidence of AKI

Author	Time Period	AKI Definition	Country	Data Source	Clinical Setting	Reported Change in Population AKI Incidence
Xue et al (2006)^20^	1992-2001	*ICD-9* AKI	United States	Medicare	All hospitalizations	Increase from 15 to 36 cases per 1000 hospitalizations
Waikar et al (2006)^21^	1988-2002	AKI-D codes *ICD-9* AKI	United States	US sample	All hospitalizations	Increase in incidence of AKI-D from 40 to 270 pmpyr Increase in incidence of AKI from 61 to 288 pmpyr
Bagshaw et al (2007)^22^	1996-2005	Creatinine and urine criteria	Australia and New Zealand	National Intensive Care Database	Intensive care admissions	Increase in incidence of AKI (4.8% vs 5.6%)
Swaminathan et al (2007)^23^	1988-2003	AKI-D codes *ICD-9* AKI	United States	US sample	Cardiopulmonary bypass	Increase in age-sex-morbidity adjusted incidence of AKI-D from 0.33% to 0.35% Increase in age-sex-morbidity adjusted incidence of AKI from 1.1% to 4.1%
Hsu et al (2007)^24^	1996-2003	AKI-D codes Creatinine change criteria	United States	Kaiser Permanente	All hospitalizations	Increase in incidence of AKI-D from 195 to 295 pmpyr Increase in incidence of AKI from 3227 to 5224 pmpyr
Thakar et al (2007)^25^	1993-2002	AKI-D Creatinine change criteria	United States	Cleveland Clinic Foundation	Cardiac surgery	Increase in incidence of AKI-D from 1.5% to 2.0% Increase in incidence of AKI from 5.1% to 6.6%
Liu et al (2010)^26^	2003-2007	AKI-D codes *ICD-10* AKI	Canada	National Discharge Abstract Database	All hospital obstetric deliveries	No change in incidence of AKI-D (40 per million deliveries) Increase in incidence of AKI from 160 to 230 per million deliveries
Callaghan et al (2012)^27^	1998-2009	*ICD-9* AKI	United States	US sample	All hospital obstetric deliveries	Increase in incidence of AKI from 229 to 452 per million deliveries
Amin et al (2012)^28^	2000-2008	AKIN creatinine change criteria	United States	Cerner Corporation Health Facts database	Acute myocardial infarction	Decrease in incidence of AKI from 26.6% to 19.7%
Siddiqui et al (2012)^29^	1995-2009	AKI-D codes	Canada	Ontario Provincial Database	All major elective surgery	Increase in incidence from 0.2% to 0.6%
Lenihan et al (2013)^30^	1999-2008	AKI-D codes *ICD-9* AKI	United States	US sample	Cardiac surgery	Increase in incidence of AKI-D from 0.45% to 1.28% Increase in incidence of AKI from 4.5% to 12.8%
Hsu et al (2013)^31^	2000-2009	AKI-D codes	United States	US sample	All hospitalizations	Increase from 222 to 533 pmpyr
Khera et al (2013)^32^	2002-2010	AKI-D codes *ICD-9* AKI	United States	US sample	Cardiac catheterization	Decrease in age-sex-morbidity adjusted incidence of AKI-D from 0.6% to 0.4% Increase in age-sex-morbidity adjusted incidence of AKI from 5.6% to 14.2%
Mehrabadi et al (2014)^33^	2003-2010	*ICD-10* AKI	Canada	National Discharge Abstract Database	All hospital obstetric deliveries	Increase in incidence of AKI from 166 to 268 per million deliveries
Sakhuja et al (2015)^34^	2000-2009	AKI-D codes	United States	US sample	Severe sepsis	Increase in incidence of AKI-D from 5.2% to 6.6%
Kolhe et al (2015)^35^	1998-2013	AKI-D codes	United Kingdom	NHS England	All hospitalizations	Increase from 15.9 to 208.7 pmpyr
Nadkarni et al (2015)^36^	2002-2011	AKI-D codes	United States	US sample	Stroke	Increase in incidence of AKI-D from 0.09% to 0.18%
Nadkarni et al (2015)^37^	2002-2010	AKI-D codes	United States	US sample	Adults with HIV	Increase in incidence of AKI-D from 0.7% to 1.35%
Nadkarni et al (2016)^38^	2006-2012	AKI-D codes	United States	US sample	Decompensated cirrhosis	Increase in incidence of AKI-D from 1.5% to 2.23%
Nadkarni et al (2016)^39^	2004-2012	AKI-D codes	United States	US sample	Adults with hepatitis C	Increase in incidence of AKI-D from 0.86% to 1.28%
Nadkarni 2016^40^	2002-2012	*ICD-9* AKI	United States	US sample	Orthopedic surgery	Increase in the incidence of AKI from 0.5% to 1.8%
Hsu 2016^41^	2007-2009	AKI-D codes	United States	US	All hospitalizations	Increase in incidence of AKI-D by 11% per year
Kolhe et al (2016)^42^	1998-2013	*ICD-10* AKI	United Kingdom	NHS England	All hospitalizations	Increase from 317 to 3995 pmpyr
Brown et al (2016)^43^	2001-2011	AKI-D codes *ICD-9* AKI	United States	US sample	Cardiac catheterization	Increase in incidence of AKI-D from 16 to 30 pmp Increase in incidence of AKI from 155 to 416 pmp
Carlson et al (2016)^44^	2000-2012	AKI-D codes	Denmark	National registry	All hospitalizations	Increase in incidence of AKI-D from 143 to 366 pmpyr
Sawhney 2017 (this article)	2001-2014	KDIGO AKI criteria *ICD-10* AKI	United Kingdom	Regional population cohort	Whole population	Increase in incidence of KDIGO AKI from 11,269 to 12,923 pmpyr Increase in incidence of *ICD-10* AKI from 663 to 2647 pmpyr

Abbreviations: AKI-D, dialysis-requiring AKI; ICD, International Classification of Diseases.

### AKI Rates Using *ICD* Coding

Over a combined period spanning 25 years, rates of hospital episode coding of non–dialysis-requiring AKI have increased in the United States,^20^, ^21^, ^32^, ^40^ Canada,^26^, ^33^ and United Kingdom.^42^ Studies from general hospital settings also report a concurrent decline in AKI mortality.^20^, ^21^, ^42^ However, wide between-study variation in AKI rates also exist (Table 1). An important limitation is that increased coding of AKI may reflect changes in coding practice more generally, changing diagnostic criteria (eg, RIFLE, AKIN, KDIGO) or increased awareness of AKI rather than a true change in disease incidence. Increased recognition of milder AKI may also explain the rising incidence in the context of falling mortality. However, it seems unlikely that an up to 10-fold increase in AKI incidence is attributable purely to changes in *ICD* coding.^21^

Similar increased rates of dialysis-requiring AKI (AKI-D) have also been reported over the last 25 years across America and Europe in settings including general hospital admissions,^21^, ^24^, ^31^, ^35^, ^41^, ^44^ elective surgery,^29^ cardiac surgery,^23^, ^25^, ^30^ coronary interventions,^43^ infectious diseases,^37^, ^39^ liver disease,^38^ obstetrics,^26^, ^27^, ^33^ sepsis,^34^ and stroke.^36^ Many of these studies also showed a declining mortality over time but not consistently.^35^ The rise in acute RRT across countries and health care settings is striking.^45^ The reasons are unclear but could include increased availability of RRT (eg, for the frail), increased recording of RRT use, financial incentives (eg, rising incidence of billing for RRT in the United States), or changing trends in medical practice (eg, a lower threshold for starting RRT). Taxation-based health care systems, such as in Denmark and the United Kingdom, have also seen increased AKI-related RRT, suggesting that financial incentives are unlikely to be the sole explanation.^35^, ^44^

### AKI Rates Using Creatinine Change Criteria

**Figure 1:**
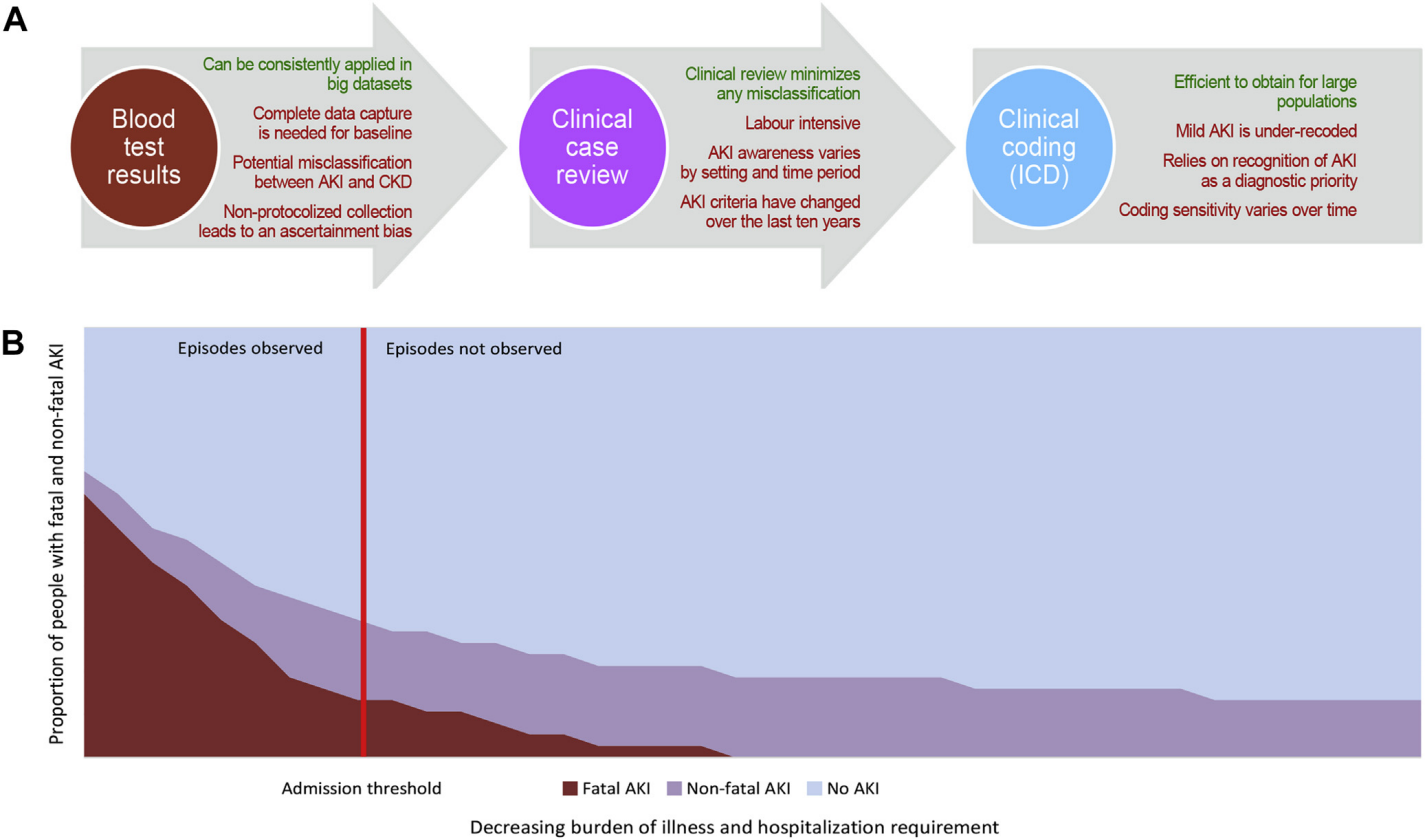
Methodologic challenges in AKI epidemiology. (A) Approaches to studying AKI using observational data and their advantages and disadvantages. (B) Bias that may arise because of convenience sampling of those admitted to hospital. In this scenario, of 1000 people in the population, 250 people had AKI (25% population incidence) including 93 who died (37% fatality). If only people above the threshold are observable, 113 people have observed AKI (11% estimated population incidence) including 80 observed deaths (71% fatality). If the admission threshold changes (eg, with a new policy), this would affect both the incidence and fatality of hospital AKI. Abbreviations: AKI, acute kidney injury; *ICD*, *International Classification of Diseases*.

The changing rates of code-classified AKI have been extensively reported, but there is a paucity of literature reporting trends in the incidence of AKI using creatinine change criteria.^22^, ^24^, ^25^, ^28^ Only 1 study has used KDIGO/AKIN–based criteria^28^ and only 1 has incorporated urine output (in an intensive care setting).^22^ Major strengths of creatinine change criteria are that they can be applied retrospectively in big data sets, capture a larger subset of AKI than that which is clinically recognized and coded,^46^, ^47^, ^48^ and enable a more consistent case ascertainment over time than is possible using *ICD* coding. In comparison, coding of AKI is dependent on clinical recognition and diagnosis, which in turn is dependent on a blood result suggesting AKI (Fig 1A). For this reason, KDIGO-based AKI criteria provide a much higher estimated incidence (10-fold) of AKI than code-classified AKI.^48^, ^49^

Nevertheless, creatinine change AKI incidence studies also have limitations, primarily relating to definitions of AKI and “baseline creatinine” (Fig 1A). Over the last decade, consensus AKI criteria have been updated 3 times.^15^, ^16^, ^50^ As each new study adopts the latest criteria, it is difficult to compare different studies, even if the definition within each study is applied consistently. Moreover, a consistently applied KDIGO-based AKI definition can only provide a representative estimate of population incidence if capture of the whole at-risk population is complete. For example, until recently, AKI that occurred and remained in the community (without admission) was under recognized.^51^ An increase in AKI observed in hospital may, therefore, reflect greater awareness and a lower threshold for hospital admission among people with AKI who were not previously admitted.^51^, ^52^ As this subgroup also have a substantially better prognosis,^52^ admission thresholds for community AKI confound many hospital AKI studies that lack complete capture of biochemistry from all clinical settings (Fig 1B). In addition, AKI can only be identified when previous (baseline) tests are available for comparison, but blood testing is clinically driven, either in acute or chronic disease management. This means that a baseline creatinine value often needs to be estimated from those previous blood tests that are available, introducing an inherent selection bias.^53^ If previous tests are infrequent and distant, then bidirectional misclassification between progressive CKD and AKI may result. Variation in the intensity of blood sampling can also introduce an ascertainment bias as more tests are generally done on sicker patients or those in a more monitored setting.^52^ Finally, if multiple tests are performed in quick succession, Lin and colleagues^54^ demonstrated through simulation that fluctuations in creatinine because of sampling variation can be sufficient to lead to a false-positive diagnosis of AKI.

Notwithstanding these limitations, 3 studies with creatinine-based definitions of AKI have reported a rising incidence of AKI in the US databases from Kaiser Permanente,^24^ Cleveland Clinic,^25^ and a national intensive care database covering Australia and New Zealand.^22^ These changes in AKI incidence were smaller (<2-fold) than in studies of code-classified AKI. In addition, 1 US study reporting AKI based on AKIN criteria after myocardial infarction actually reported fall in AKI incidence and mortality,^28^ which was attributed to improved clinician awareness, care and prevention of AKI, and may also relate to case selection.

### AKI Rates Using *ICD-10* Coding and KDIGO-Based Criteria Simultaneously

**Figure 2:**
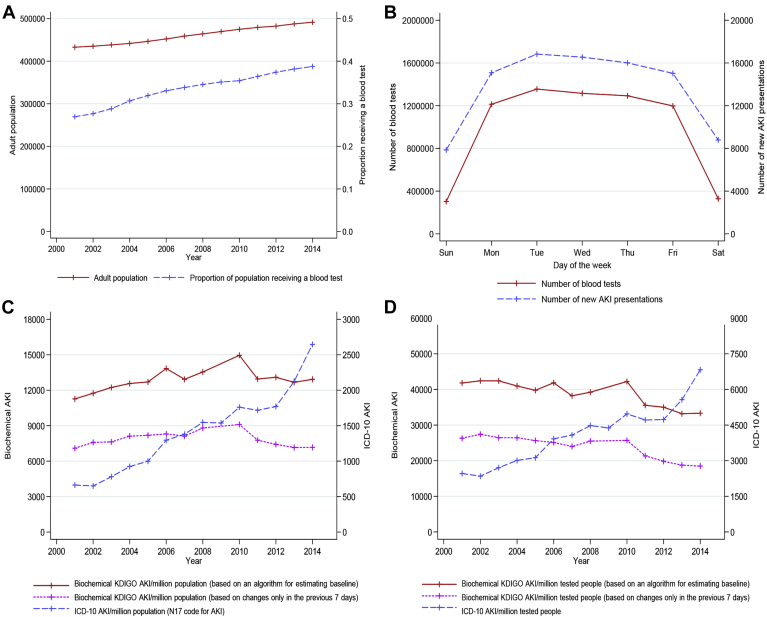
Study of the incidence of AKI in the Grampian population 2001-2014. (A) Growth of Grampian population (red solid) and increase in the proportion of people receiving a blood test (blue dash). (B) Association between testing intensity and the incidence of new AKI presentations by day of the week 2001-2014. (C) Rates of KDIGO-AKI using creatinine change criteria (red solid and pink dot) and ICD-10 code-classified AKI (blue dash). (D) AKI incidence represented as a proportion of the tested population at risk. Abbreviations: AKI, acute kidney injury; *ICD-10*, *International Classification of Diseases, Tenth Revision*; KDIGO-AKI, Kidney Disease: Improving Global Outcomes.

Although studies suggest that AKI and RRT use are increasing, the limitations of *ICD* coding accuracy and biochemistry testing intensity mean that true trends in population AKI remain hard to interpret. To illustrate the influence of these methodologic issues with AKI identification, in Figure 2A-D, we have described the incidence of AKI using all biochemistry in the Grampian (UK) population (*n* = 500,000). We have contrasted *ICD-10* code-classified AKI (N17), KDIGO-based AKI (first, estimating baseline as described by Sawhney and colleagues^7^ and second, estimating baseline only using blood tests strictly taken in the past week), and the intensity of biochemistry sampling. Those already receiving long-term RRT were excluded as previously described.^7^ Although the Grampian population has grown slowly over 14 years, there has been a disproportionate increase in the intensity of biochemical testing; 39% of the population now receive at least 1 blood test/year (Fig 2A) compared with 27% in 2001. In addition, although KDIGO-AKI has increased slowly 2001-2014 (from 11,269 to 12,923 per million population/year, pmpyr), code-classified AKI has quadrupled over the same period (from 663 to 2647 pmpyr) (Fig 2C). If AKI were reported as a ratio of cases to the number of tested individuals at risk, the rate of KDIGO AKI could actually be considered to have fallen (from 41,816 to 33,337/million people receiving blood tests) (Fig 2D). Ascertainment bias because of testing intensity is also suggested by a crude comparison of the day of the week on which AKI episodes initially present vs number of tests performed on each day (Fig 2B).

## The Long-term Prognosis of AKI

An association between AKI and poor long-term outcomes is supported by evidence from systematic reviews of over 50 studies linking AKI with increased mortality, CKD, and long-term RRT.^55^, ^56^, ^57^ Accordingly, KDIGO AKI guidelines advocate follow-up of patients after hospital discharge.^16^ Biologic plausibility is also supported by basic science models^58^ and evidence from extended follow-up of kidney transplant donors revealing increased long-term RRT requirement.^59^ However, given the high incidence of hospital AKI and the fact that only a minority see a nephrologist after discharge,^60^ there is a need to help clinicians prioritize those with the greatest risk and the most modifiable risk of poor outcomes. Large observational studies can inform this clinical need because subgroup analyses are possible with adequate power. Several large population and hospital-based cohort studies from Canada, the US Veteran Affairs (VA) health system, and the UK National Health Service have evaluated outcomes of AKI within subgroups of baseline kidney function, AKI severity, renal recovery, and follow-up time.

### AKI Outcomes Grouped by Baseline CKD and Follow-up Time

In 864,933 VA patients including 82,711 meeting AKIN criteria for AKI, Lafrance and colleagues^61^ reported AKI mortality in baseline subgroups over 2.4 years. AKI mortality hazard ratios (HRs) were greater among those with a higher baseline estimated glomerular filtration rate (eGFR) than a lower baseline eGFR: HRs 1.45, 1.35, 1.31, and 1.23 for eGFR ≥90, 60-89, 45-59, and 30-44 mL/min/1.73 m^2^, respectively. In 29,388 separate VA individuals undergoing cardiac surgery, Ishani and others grouped patients according to the severity of postoperative creatinine rise (≤0%, 1-24%, 25-49%, 50-99%, and ≥100%) and in time intervals at risk to determine if mortality, new CKD, and long-term RRT varied over follow-up time. This concept of a varying excess risk following an exposure is familiar in renal transplantation research,^62^ where an insult (eg, transplant procedure) may initially be detrimental before later yielding benefit, but the application in AKI was novel. The investigators found outcomes were poorer among those with greater postoperative creatinine increase, but even small rises in creatinine were associated with poor renal outcomes. Although the excess risk of AKI was greatest over the first year after hospitalization, it persisted up to 5 years.^63^ A limitation was that this study was limited to AKI in a cardiac setting, which may not be generalizable. In addition, both studies were conducted in cohorts of older US veterans with few women.

**Figure 3:**
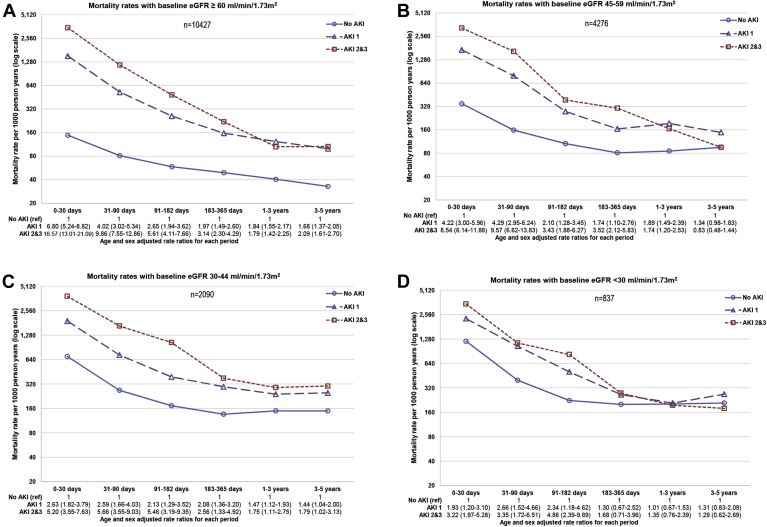
Mortality rates and age- and sex-adjusted rate ratios by (A-D) baseline eGFR group and acute kidney injury (AKI; 1-3 denote severity stage). Abbreviations: AKI, acute kidney injury; ref, reference group; eGFR, estimated glomerular filtration rate. Note the log scale: each increment on the *y* axis represents a doubling of mortality rates.

Modified from Sawhney et al.,^7^http://dx.doi.org/10.1053/j.ajkd.2016.05.018 under the terms of the Creative Commons Attribution License (CC BY).

The concurrent interactions of baseline CKD and follow-up time on the relationship between AKI and death were recently tested together in a single general population study of 17,630 hospital admissions with and without AKI in the UK.^7^ The authors separated outcomes by baseline eGFR, AKI severity, and short (0-30 days), intermediate (31-364 days), and long-term (1-10 years) time periods for those at risk. Consistent with the 2 previous Veteran Affairs studies, over 10-year follow-up mortality was greatest among those with AKI and severe baseline CKD (eGFR <30 mL/min/1.73 m^2^), and the excess risk from AKI was greater among those with a higher baseline eGFR than with a lower baseline eGFR (who were already at elevated risk irrespective of AKI) (Fig 3A-D). Excess risk of AKI and AKI severity also diminished over time, such that for patients with baseline eGFR <30 mL/min/1.73 m^2^, even a severe AKI episode no longer conferred additional mortality risk among people who had already survived 1 year. This study also identified previous AKI episodes as an additional adverse prognostic factor in those with AKI (37% greater long-term mortality). However, the primary outcome was mortality and progression of CKD not reported.^7^

### AKI Outcomes Grouped by Renal Recovery

Further large database subgroup analyses of AKI outcomes have focused on renal recovery to baseline as a stratifying risk factor. In a population study of hospitalizations in Alberta, Pannu and others^64^ compared people with AKI who subsequently recovered to within 25% of baseline and people with AKI who did not recover. Over a median 34 months, those who did not recover had increased mortality (adjusted HR 1.26, 95% confidence interval [CI] 1.10-1.43) and renal outcomes (HR 4.13, CI 3.38-5.04). A feature of this study was a detailed sensitivity analysis of different thresholds for defining renal recovery, with the worst outcomes among those with little or no recovery. A further US single-center study propensity matched 1610 people hospitalized with AKI who recovered to within 90% of baseline (vs hospitalization without AKI). Over a median 3.3 years, AKI patients with near-complete recovery still had increased mortality (HR 1.5, CI 1.21-1.85) and *de novo* CKD (HR 1.91, 1.75-2.09).^65^ However, this was a single-center study and findings may not be generalizable. The authors also did not account for changes in the HR over follow-up time.

### Implications and Limitations

Collectively, these studies provide evidence of greater mortality and adverse renal outcomes after AKI, with baseline eGFR, previous episodes, renal recovery extent, and follow-up time as key factors on which to further risk stratify and ensure those whose risk of poor outcomes have increased are sufficiently monitored. However, the consequences of new models of care should be evaluated, and we note that a dedicated post-AKI clinic is currently under evaluation in Canada.^66^ The greater excess risk of AKI among those with normal baseline kidney function is perhaps counterintuitive, but those who have baseline CKD are already at high risk and may share many confounding risk factors (measurable and unmeasurable) that predispose toward AKI, CKD, and mortality.

For the clinician, the main implication is that any form of renal impairment, acute or chronic, is associated with poorer outcomes, but the excess of poor outcomes after AKI are observed mainly in the first year after hospitalization. Whereas early reassessment could be guided by the severity of the acute illness and extent of recovery, long-term monitoring could be guided by chronic factors, such as new or worsening chronic kidney disease.

These observational studies using creatinine change criteria also have important limitations. Causality cannot necessarily be inferred from these observational studies. Even high-quality cohort studies are subject to risk of bias and confounding, and causality is, therefore, contested.^9^, ^10^ On the other hand, many relevant randomized trial designs would be unethical. Moreover, bidirectional misclassification of AKI and CKD due to incomplete baseline data means that some renal outcomes attributed to AKI may actually represent rapidly progressing CKD. Variation in blood testing intensity across clinical settings and time will influence the extent of CKD misclassification and the extent of this bias. Similarly, as previously described, variation in data capture of people with AKI in the community will skew the impression of the overall burden and distribution of AKI. Those who meet KDIGO AKI criteria and are not promptly admitted to hospital have a substantially better short-term prognosis, which means that failure to account for this subgroup will adversely affect overall AKI prognosis and incidence estimates.^52^ Admission thresholds may also vary between health care settings and over time. Convenience sampling in AKI studies may lead to bias, with a focus on severe, high-mortality AKI, and under-ascertainment of milder AKI cases with a low mortality. As a result, the full population burden is underestimated, and the case fatality rate is overestimated (Fig 1B). Notably, similar sampling bias can also occur in studies limited to a critical care setting or restricted to code-classified AKI. Finally, studies of renal recovery should also be interpreted carefully due to the effect of critical illness on muscle mass and accordingly creatinine assays. A study of 700 National Health Service intensive care survivors reported discharge creatinine values below baseline for those without AKI. Adjusting for this confounding effect of major illness led to an estimated 135% increase in post-discharge CKD diagnoses.^67^

## The Role of Large Databases and AKI in Care Quality

Following a UK enquiry into patient outcome and death (National Confidential Enquiry into Patient Outcome and Death) from AKI, there was international recognition of the need to improve care.^17^, ^68^ Given the poor outcomes after AKI across all clinical settings,^8^ interest has focused on interventions that may improve recognition and early management. Initiatives include AKI e-alerts and care bundles which are covered in a later article within this issue. The “Tackling Acute Kidney Injury” trial is notable because it will use data linkage to follow-up outcomes in 5 participating hospitals.^69^ However, other approaches to quality improvement use large databases to monitor policy changes and initiatives. This is illustrated by 2 quazi-experimental studies using interrupted time-series analysis.^70^, ^71^

Bell and colleagues monitored the consequences of a regional policy change from co-amoxiclav to gentamicin prophylaxis for non-neck of femur (non-NOF) orthopedic procedures as part of a target to reduce *Clostridium difficile* in Scotland. They assessed the rates of *C. difficile* (an intended consequence) and of AKI (an unintended consequence). NOF procedures were a control group where policy did not change.

**Figure 4:**
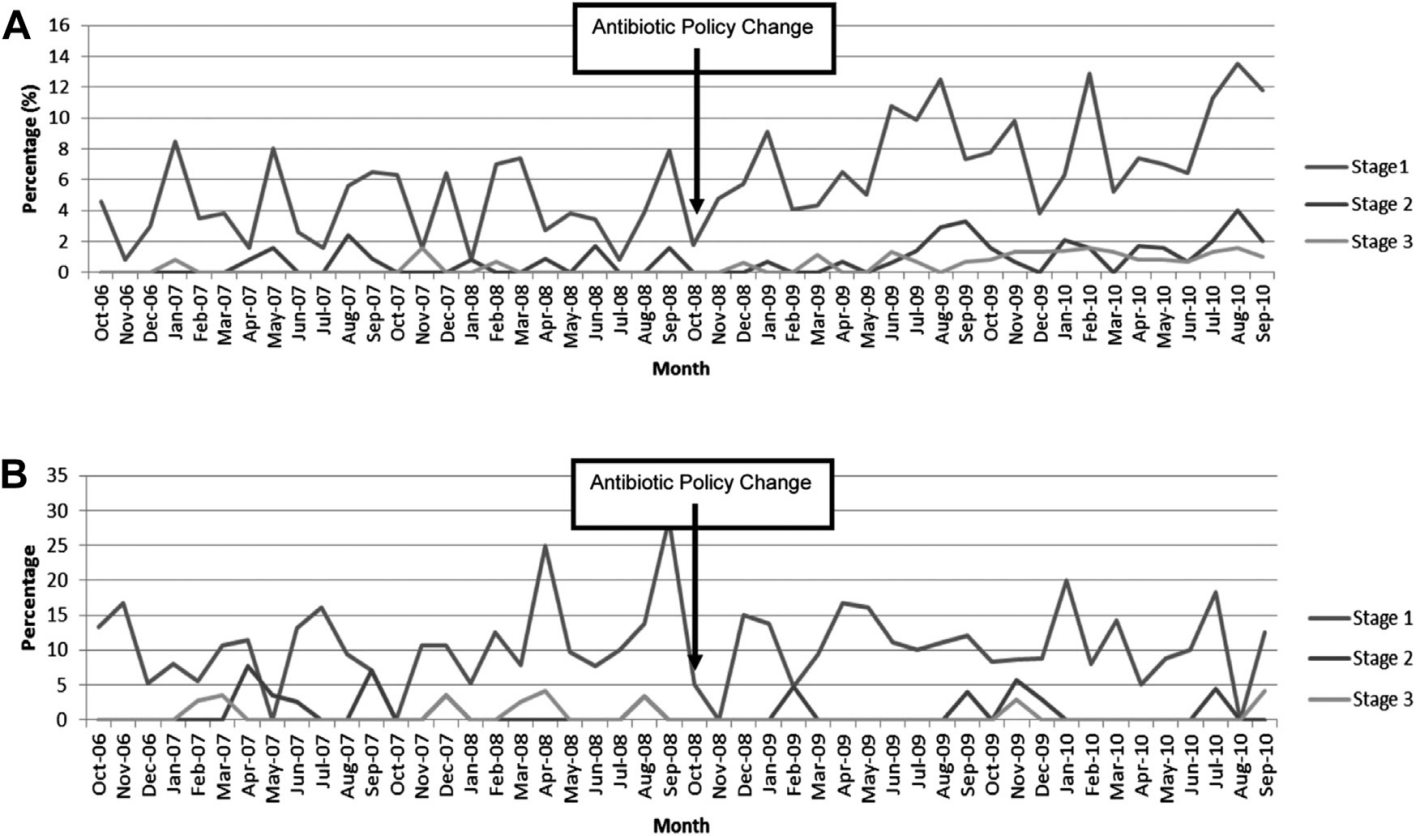
Percentage of people undergoing surgery who developed postoperative AKI stages 1, 2, and 3: (A) following a gentamicin policy change among people undergoing orthopedic surgery (excluding NOF). (B) People undergoing surgery of an NOF fracture (for whom the policy change did not involve gentamicin). Abbreviations: AKI, acute kidney injury; NOF, non-neck of femur.

Reproduced from Bell et al.,^70^http://dx.doi.org/10.1681/ASN.2014010035, with permission from the authors and publisher.

The gentamicin policy did not reduce *C. difficile* rates but caused an unintended 94% increase in KDIGO AKI in non-NOF procedures (Fig 4A) compared with no change in NOF procedures (Fig 4B).^70^ This increase in AKI subsequently reduced 63% when the policy was reversed.^71^ This demonstrates the power of routine data for monitoring intended and unintended consequences of health care changes and inform new policy.

## Conclusion

Large database studies show that AKI pervades health care systems worldwide, with poor outcomes and an increasing use of RRT. Given the high incidence of AKI hospital survivors, prognostic risk stratification on AKI severity, baseline, recovery to baseline, and follow-up time, may be helpful to improve post-AKI care, but such an approach would need careful evaluation. Temporal and regional comparisons of AKI incidence and prognosis may be biased by changes in AKI awareness, monitoring, recognition, and clinical practice. For this reason, researchers monitoring quality initiatives using observational data should be careful to capture all who may be affected by policy changes and ensure that both intended and unintended consequences are recognized.
